# ARTAG in the basal forebrain: widening the constellation of astrocytic tau pathology

**DOI:** 10.1186/s40478-016-0330-7

**Published:** 2016-06-13

**Authors:** Alan King Lun Liu, Marc H. Goldfinger, Hayleigh E. Questari, Ronald K. B. Pearce, Steve M. Gentleman

**Affiliations:** Neuropathology Unit, Division of Brain Sciences, Department of Medicine, Imperial College London, Burlington Danes Building, Hammersmith Hospital Campus, London, W12 0NN UK

**Keywords:** Chronic traumatic encephalopathy, Aging-related tau astrogliopathy, Basal forebrain, Tauopathy

Tauopathies are disorders characterised by the abnormal accumulation of hyperphosphorylated tau protein within neurons and glial cells. Alzheimer’s disease (AD), the most common form of tauopathy, features a stereotypical, staged progression of neurofibrillary tangle (NFT) and neuritic tau pathology through the brain [1]. However, age-related NFT and neuritic tau pathologies have also commonly been identified in cognitively normal and ‘tangle-only dementia’ cases in the absence of amyloid-β peptide (Aβ) pathology. This has now been given the term ‘primary age-related tauopathy’ (PART) to distinguish it from AD [3]. Furthermore, astroglial tau aggregations have been increasingly recognised to be present within the aging brain independently of any co-existing neuropathological disorders or cognitive impairment. This unique astroglial tau pathology has been termed aging-related tau astrogliopathy (ARTAG) [6]. ARTAG exists in two distinct morphological forms as thorn-shaped astrocytes (TSA) and granular/fuzzy astrocytes (GFA). In addition, these tau immunoreactive astrocytes show a unique distribution within the brain, with TSA and/or GFA commonly found in subpial, subependymal and perivascular locations, as well as in clusters within the white and grey matter.

In a large population-based cohort study, Wharton and colleagues attempted to elucidate the relationship between astroglial tau pathology and neuronal tau and to correlate it with dementia status in aged brains [12]. Although no significant correlation was identified, astroglial tau pathology was found to be prevalent, with TSA present in 40 % of the cases. In particular, the authors mentioned that TSA are commonly found in a subpial location inferior to the substantia innominata but no detailed quantitative or morphological assessment was described.

As part of our ongoing research related to basal forebrain pathology in Lewy body disorders (LBD), we noted incidental ARTAG pathology in a significant number of cases. We surveyed 702 consecutive cases from the Parkinson’s UK Tissue bank at Imperial College London and 83 cases from Brains for Dementia Research (BDR) tissue bank from Newcastle University, excluding those with incomplete clinical histories, vascular pathology and significant co-existing neuropathological comorbidities. We selected 199 cases with a neuropathological diagnosis of pure LBD (BrainNet Europe Braak tau stage <4) or AD together with 26 age-matched controls. Basal forebrain sections containing the nucleus basalis of Meynert were immunostained for tau (AT8) and 76 cases (33.8 % of total) were found to display ARTAG. Our cohort is different from that in the Wharton et al study in that the mean age of our cohort is younger (78.8 vs 85.9) and we have a greater number of LBD cases compared with their study. Out of 155 cases with a diagnosis of Parkinson’s disease (PD), 48 (31.0 %) displayed ARTAG pathology in the basal forebrain, compared with 25.0 % in dementia with Lewy bodies (DLB) cases, 41.7 % in AD cases and 50.0 % in controls (Table 1). However, ARTAG within the basal forebrain does not seem to correlate with cognitive decline as 29 out of 78 cases (37.2 %) without cognitive impairment showed ARTAG compared with only 47 out of 147 (32.0 %) in those with cognitive impairment (Chi-square test *p* = 0.432), and the two groups are age-matched (mean age 78.69 vs 78.90, *t*-test *p* = 0.854). ARTAG pathology appeared to be more common in males (65.8 % of ARTAG-positive cases) although this did not reach statistical significance (Chi-square test *p* = 0.082). Consistent with the existing literature, the presence of ARTAG pathology increases with age, from the age of 60 (Table 2). As a result, the seemingly higher prevalence of ARTAG pathology in control and AD cases may be due to the higher age at death (mean = 87.75) compared with PD (mean age at death = 77.25) and DLB cases (mean age at death = 79.55). The perivascular distribution of ARTAG pathology is of particular interest because a weakening of the blood-brain barrier has also been associated with increasing age [11].

**Table 1:** Demographics and presence of ARTAG pathology of screened cases

Primary neuropathological diagnosis	Control	Parkinson’s disease	Dementia with Lewy bodies	Alzheimer’s disease
n	26	155	20	24
M:F (% male)	8:18 (30.8 %)	98:57 (63.2 %)	13:7 (65.0 %)	11:13 (45.8 %)
Mean age at death (SD)	81.35 (10.159)	77.25 (7.685)	79.55 (6.160)	85.75 (5.848)
Cases with presence of ARTAG in basal forebrain (%)	13 (50.0 %)	48 (31.0 %)	5 (25.0 %)	10 (41.7 %)

**Table 2:** Presence of ARTAG pathology among different age groups

Age group	Cases with presence of ARTAG (%)	Cases with absence of ARTAG (%)
50–59	0 (0.0 %)	4 (100.0 %)
60–69	7 (25.9 %)	20 (74.1 %)
70–79	21 (28.0 %)	54 (72.0 %)
80–89	40 (38.5 %)	64 (61.5 %)
90–99	8 (53.3 %)	7 (46.7 %)

Morphologically, astrocytic tau pathology seen in our cohort was distinct from the tufted astrocytes seen in progressive supranuclear palsy [13] and LBD [4], and the astrocytic tau-positive inclusions in corticobasal degeneration and Pick’s disease [5]. 67 cases (88.16 %) had only TSA, 3 (3.95 %) had GFA and 6 had both (7.89 %) TSA and GFA. In terms of distribution of pathology, ARTAG in the basal forebrain is most commonly found perivascularly (42.53 %), and subpially (34.48 %) (Fig. 1, Additional file 1: Table S1). However, within the anterior basal forebrain (at the anterior commissure decussation level, as previously delineated [8]), ARTAG is most prevalent in the subpial location (39.13 %) followed by a perivascular distribution (30.43 %). Using the severity of deposition parameters suggested by Kovacs et al [6], ARTAG was most commonly identified focally at the intermediate (54.05 %) and posterior (51.61 %) levels of the basal forebrain, but only occasionally at the anterior level (41.38 %) of the basal forebrain.

**Fig. 1:**
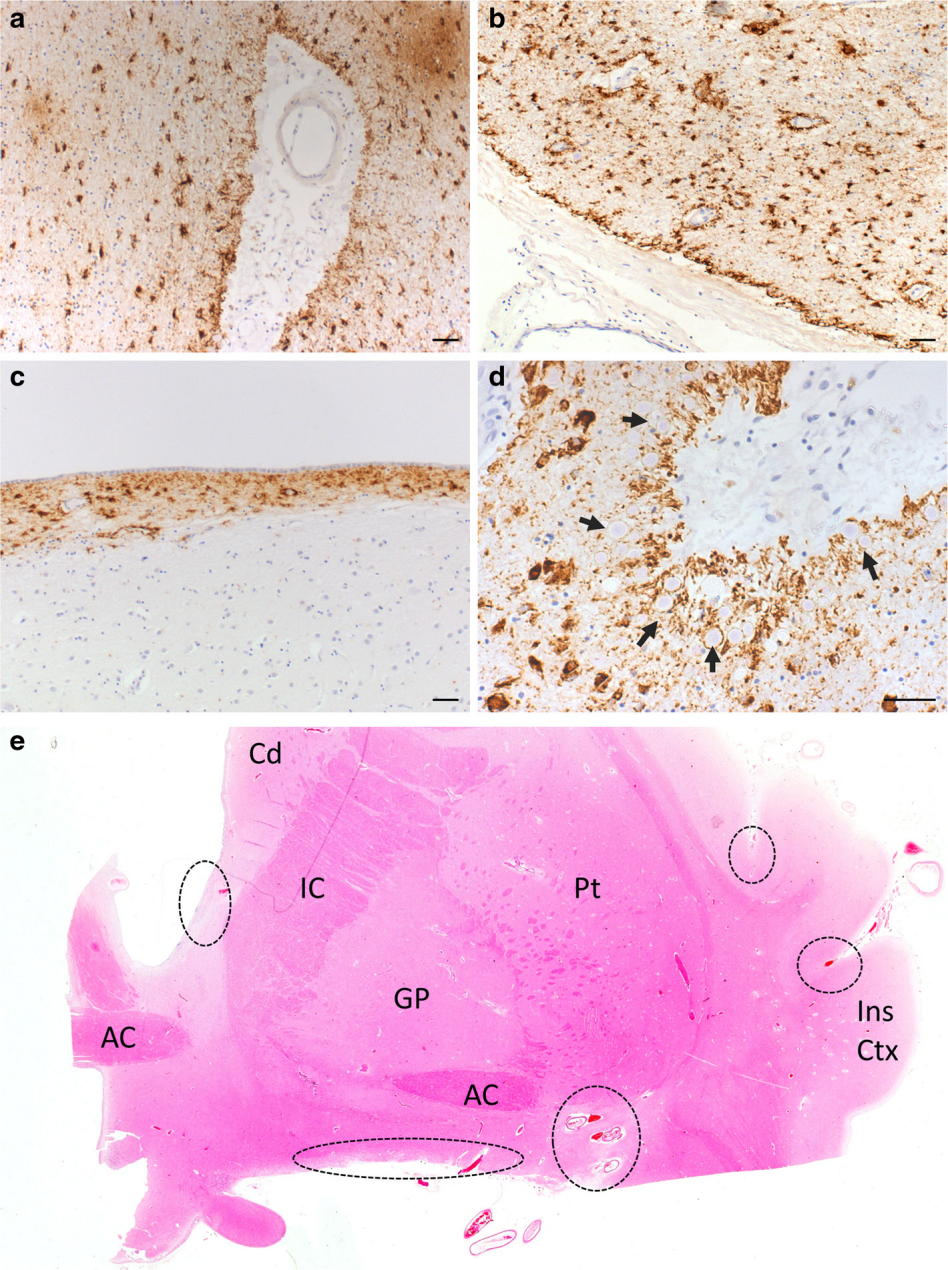
Characteristic ARTAG astrocytic tau pathology seen in the basal forebrain around large vessels (**a**,**b**), in the subpial area at the ventral surface of the brain (**b**) and periventricularly (**c**). Corpora amylacea around large vessels in close proximity to astrocytic tau pathology. Arrows pointing to corpora amylacea (**d**). Scale bars = 50 μm for (a-d). H&E image of the human basal forebrain (adapted from [8]) with common areas for ARTAG tau pathology outlined in dashed circles (**e**). Abbreviations: AC, anterior commissure; Cd, caudate; GP, globus pallidus; IC, internal capsule; Ins Ctx, insula cortex; Pt, putamen

ARTAG pathology in the aging brain shares a similar distribution and morphology to that seen in chronic traumatic encephalopathy (CTE), a neurodegenerative sequala of repeated traumatic brain injury [9]. Co-morbid neurodegenerative disorders have also been identified within CTE cases [10]. Within a large cohort of neurodegenerative diseases and controls, Ling et al reported evidence of CTE pathology in 32 out of 268 (11.9 %) screened cases and in 12.8 % of age-matched controls, and 30 (93.8 %) of CTE-positive cases had history of traumatic brain injury [7]. However, in another study where CTE pathology was screened in cortical brain sections of 198 controls and 66 individuals with a history of involvement in contact sports [2], CTE pathology was only identified in 21 of the 66 individuals at risk but not in the control group. This conflicting finding could be due to the different age ranges of the cases screened in these two studies. The 32 CTE-positive cases identified in the Ling et al study were all over the age of 60 (mean age = 80.97), and the 6 CTE-positive control subjects had a mean age of 92.17 which is considerably higher than the all cases screened in the Bieniek et al study (mean age ~75). This suggests that CTE and ARTAG possibly share a common aetiological pathway. The predominance of ARTAG pathology in males in our cohort is similar to that seen in CTE. Detailed retrospective clinical note analysis was not performed but a number of cases had a recorded history of head injury, participation in contact sport or military service. Furthermore, CTE pathology may reflect advanced aging or injury induced ARTAG or both. Wharton and colleagues noted the presence of corpora amylacea in close proximity to astroglial tau accumulations [12], and we report a similar observation in the basal forebrain (Fig. 1d). The accumulation of corpora amylacea is hypothesised to be involved in a disposal pathway of tau aggregates, which might further explain the close proximity of these bodies to tau pathology. Further work in a large cohort is needed to examine the relationship between CTE and ARTAG pathology throughout the brain and further clinicopathological research will add to our understanding of the pathogenesis of CTE and ARTAG in relation to the process of normal aging and neurodegenerative disorders.

## Ethical considerations

The work conducted on human tissue was under ethical approval held by the Parkinson’s UK Brain Bank at Imperial College London (Registered charity in England and Wales (258197) and in Scotland (SC037554); Multicentre Research Ethics Committee approval reference number: 07/MRE09/72). Parkinson’s UK Brain Bank is an approved Research Tissue Bank by the Wales Research Ethics Committee (Ref. No. 08/MRE09/31 + 5). Further tissues were provided by the Newcastle Brain Tissue Resource, which is funded in part by a grant from the UK Medical Research Council (G0400074) and by Brains for Dementia research, a joint venture between Alzheimer’s Society and Alzheimer’s Research UK.

## Additional file

Additional file 1: Additional information on case demographics and qualitative/semi-quantitative assessment of ARTAG pathology with summary of data. ARTAG pathology was semi-quantitatively rated 1-4 (low to high burden) under columns K-U. (XLSX 30 kb)

## Abbreviations

AD: Alzheimer’s disease
ARTAG: Aging-related tau astrogliopathy
BDR: Brains for Dementia Research
CTE: Chronic traumatic encephalopathy
DLB: Dementia with Lewy bodies
GFA: Granular/fuzzy astrocytes
LBD: Lewy body disorders
NFT: Neurofibrillary tangle
PART: Primary age-related tauopathy
PD: Parkinson’s disease
TSA: Thorn-shaped astrocytes
